# The short-term efficacy and safety of artificial total disc replacement for selected patients with lumbar degenerative disc disease compared with anterior lumbar interbody fusion: A systematic review and meta-analysis

**DOI:** 10.1371/journal.pone.0209660

**Published:** 2018-12-28

**Authors:** Xiaoping Mu, Jianxun Wei, Jiancuo A., Zhuhai Li, Yufu Ou

**Affiliations:** 1 Department of Orthopaedic, The People’s Hospital of Guangxi Zhuang Autonomous Region, Nanning, China; 2 Department of Spinal surgery, Qinghai Red Cross Hospital, Xining, Qinghai, China; Universita degli Studi di Palermo, ITALY

## Abstract

**Purpose:**

To systematically compare the efficacy and safety of lumbar total disc replacement (TDR) with the efficacy and safety of anterior lumbar interbody fusion (ALIF) for the treatment of lumbar degenerative disc disease (LDDD).

**Methods:**

The electronic databases PubMed, Web of Science and the Cochrane Library were searched for the period from the establishment of the databases to March 2018. The peer-reviewed articles that investigate the safety and efficacy of TDR and ALIF were retrieved under the given search terms. Quality assessment must be done independently by two authors according to each item of criterion. The statistical analyses were performed using RevMan (version 5.3) and Stata (version 14.0). The random-effect model was carried out to pool the data. The *I*^2^ statistic was used to evaluate heterogeneity. The sensitivity analysis was carried out to assess the robustness of the results of meta-analyses by omitting the articles one by one.

**Results:**

Six studies (5 randomized controlled trials (RCT) and 1 observational study) involving 1093 patients were included in this meta-analysis. The risk of bias of the studies could be considered as low to moderate. Operative time (MD = 4.95; 95% CI -18.91–28.81; P = 0.68), intraoperative blood loss (MD = 4.95; 95% CI -18.91–28.81; P = 0.68), hospital stay (MD = -0.33; 95% CI, -0.67–0.01; P = 0.05), complications (RR = 0.96; 95% CI 0.91–1.02; P = 0.18) and re-operation rate (RR = 0.54; 95% CI 0.14–2.12; P = 0.38) were without significant clinical difference between groups. Patients in the TDR group had higher postoperative satisfaction (RR = 1.19; 95% CI 1.07–1.32; P = 0.001) and, better improvements in ODI (MD = -10.99; 95% CI -21.50- -0.48; P = 0.04), VAS (MD = -10.56; 95% CI -19.99- -1.13; P = 0.03) and postoperative lumbar mobility than did patients in the ALIF group.

**Conclusions:**

The results showed that TDR has significant superiority in term of reduced clinical symptoms, improved physical function and preserved range of motion for the treatment of LDDD compared to ALIF. TDR may be an ideal alternative for the selected patients with LDDD in the short-term. However, the results of this study cannot suggest the use of TDR instead of ALIF in lumbar spine treatment only in the light of short term results. More studies that are well-designed, that are of high-quality and that have larger samples are needed to further evaluate the efficacy and safety of TDR with at the long-term follow-up.

**Level of evidence:**

Therapeutic Level 3

## Introduction

Chronic low back pain (CLBP) is generally defined as lower back pain that persists for at least 3 months or 12 weeks [1]. More than 80% of all individuals experienced at least 1 episode of LBP at some point in their lifetime [2]. Obviously, the impact of LBP is substantial, not only on the individual, but also on communities and health systems [3,4]. Current research is still not clear about the causes of CLBP, but lumbar disc degenerative disease is considered to be closely related to CLBP.

For the patients seeking medical intervention for CLBP, lumbar operation will be considered when conservative treatments are ineffective for 6 months. ALIF, which was first reported by Capener in 1932, has developed into a mature and popular operative method for the treatment of LDDD. However, as an important part of many different fusion approaches, ALIF is not perfect. It not only has some complications similar to other fusion approaches, such as adjacent segment degeneration [5] and postoperative fusion pain, but is also associated with the risk of vascular, intestinal and nerve injury [6]. Although those complications associated with the approach can be decreased through improving techniques and continuous training, the restriction in the range of motion (ROM) of the fusion segment generally cannot be changed.

As an alternative to lumbar fusion, artificial lumbar total disc replacement (TDR), which is continuously applied in clinical practice, can not only eliminate the adverse effects on spinal stability after discectomy by restoring and preserving normal inter-segmental motion [7,8], but also avoid the non-physiological load of adjacent segments to slow down degeneration [9]. Biomechanical study has already demonstrated that TDR can maintain the three-dimensional motion and restore the biomechanical properties of lumbar vertebrae [10]. Relevant clinical studies have been reported, but it is not clear which one is better or worse in the efficacy of TDR and ALIF.

Scholars [11–14] have carried out several meta-analysis to survey the safety and efficacy of TDR for patients with LDDD compared with lumbar fusion. However, the application of different fusion approaches in various studies may lead to certain bias and reduce the level of evidence after pooling data for the same outcome. ALIF and TDR have similar anatomical approaches, and there are several clinical studies comparing TDR and ALIF in the treatment of LDDD. Therefore, we believe that it is necessary to perform a detailed stratification of lumbar fusion in a new meta-analysis on comparison of TDR and ALIF.

## Materials and methods

This systematic review and meta-analysis was performed according to the Preferred Reporting Items of systematic review and meta-analysis (PRISMA) items [15]. (S1 File)

### Search strategy

Two authors (M.XP and W.JX) who have been educated in literature retrieval courses independently searched electronic databases including PubMed, Web of Science and the Cochrane Library for the period from the establishment of the databases to March 2018. The following search terms were used: (“total disc replacement” OR “lumbar disc arthroplasty” OR “artificial disc replacement”) AND (“anterior lumbar interbody fusion” OR “anterior fusion” OR “ALIF”). Due to the limitations of the authors’ own language, the studies written in other languages other than English and Chinese, were not considered for the inclusion in the present study. The list of references of the relevant review and the included studies were also further hand-checked one by one to identify studies that had not been retrieved in the preliminary database search.

### Selection criteria

The eligibility criteria in the present study included the following: (1) study design: randomized control trials and observational studies; (2) case population: adult patients with LDDD underwent lumbar TDR or ALIF; (3) intervention methods: TDR (investigative group) versus ALIF (control group); and (4) outcome measures: study containing at least one of the desired evaluation indicators for this meta-analysis. Exclusion criteria were formulated as follows: (1) a study containing patients with the history of lumbar surgery prior to TDR/ALIF; (2) a case report, animal experiment, or biomechanical research paper; and (3) a study reporting patients who underwent combinations of both of the interventions (TDR or ALIF) and/or one of the interventions and other surgical procedures.

### Data extraction

Two authors (M.XP and O.YF) independently extracted the data in accordance with the established criteria and filled in the standardized form immediately. The following information was extracted from every study: (1) study characteristics: authors, publication year, study design, and number of patients each group; (2) surgical data: operative time, estimated blood loss, and duration of hospitalization; (3) curative effect evaluation: VAS scores, ODI scores, and patient satisfaction; (4) radiological parameter: range of motion (ROM) of each lumbar segment; and (5) postoperative complications and re-operation: infections, approach-related events, and neurological events, among other complications.

### Risk of bias assessment and quality of evidence

Three reviewers (M.XP, L.ZH and W.JX) independently used the bias risk assessment tool provided by Cochrane back review group [16] for RCTs and the Newcastle-Ottawa Quality Assessment Scale (NOQAS) [17] for cohort and case-controlled studies to evaluate the quality of each study. Each RCT was re-examined and “yes”, “no” or “unsure” were answered provided for in the following items: (1) random sequence generation, allocation concealment and baseline similarity for the important prognostic indicators (selection bias); (2) blinding of patients and care provider, co-interventions (performance bias); (3) acceptable drop-out rate and analysis of randomized participants (attrition bias); (4) blinding of outcome assessor and identical timing of outcome assessment (measurement bias); (5) suggestion of selective outcome reporting (reporting bias); and (6) other: other sources of potential bias. NOQAS was composed of nine items in the following three categories: selection of the study population, comparability among groups and outcome evaluation for cohort studies or exposure for case-controlled studies. Out of a total score of 9 points, studies with less than 5 points were considered as low quality studies; studies with 5 points or greater were rated as high quality studies. The Grading of Recommendations Assessment, Development and Evaluation (GRADE) system [18] was used by two reviewers (M.XP and O.YF) to assess the confidence in effect estimates. The quality of evidence was considered as high, moderate, low or very low involving the following domains: risk of bias, inconsistency, indirectness, imprecision, and publication bias. When disputes between the reviewers regarding results could not be resolved through their internal negotiation, new reviewers would be added.

### Date analysis and interpretation

An open and free statistic software, RevMan 5.3 version and Stata 14.0, were downloaded and synthesized for all of available data in these relevant studies. Meta-analysis and forest plots were expected to construct. Mean difference (MD) was used for continuous outcomes with identical scales. Otherwise, we used standardized mean difference (SMD). The relative risks (RRs) were carried out to analysis for binary outcomes. the corresponding 95% confidence interval (95% CI) was provided for each outcome. The random-effect (RE) model was carried out to pool the data, as this model is more conservative and provides better estimates with wider confidence intervals than the fixed-effects model. The *I*^2^ statistic was used to evaluate heterogeneity; values of 25%, 50%, and 75% were considered low, moderate, and high heterogeneity, respectively [19]. The presence of heterogeneity warrants examining the sources where we used covariates in a meta-regression analysis. In this analysis, the covariates were used were the following: sample size (<200 cases vs. >200 cases), study design (prospective vs. retrospective), and follow-up points (<24 months vs. >24 months). The publication bias was not evaluated, as a small meta-analysis normally under-powered to detect much bias and this analysis tends to lead to conclusions that are not justified. The sensitivity analysis was carried out to assess the robustness of the results of meta-analyses by omitting the articles one by one. Under the same evaluation index, an inappropriate data format from the included studieswas not pooled with this software. But, to avoid missing meaningful results and to minimize the bias of the report, these data were still displayed reported in the results section by descriptive text. A double-sided p value less than 0.05 was considered as a significant difference.

## Results

### Literature search and study characteristic

**Fig 1:**
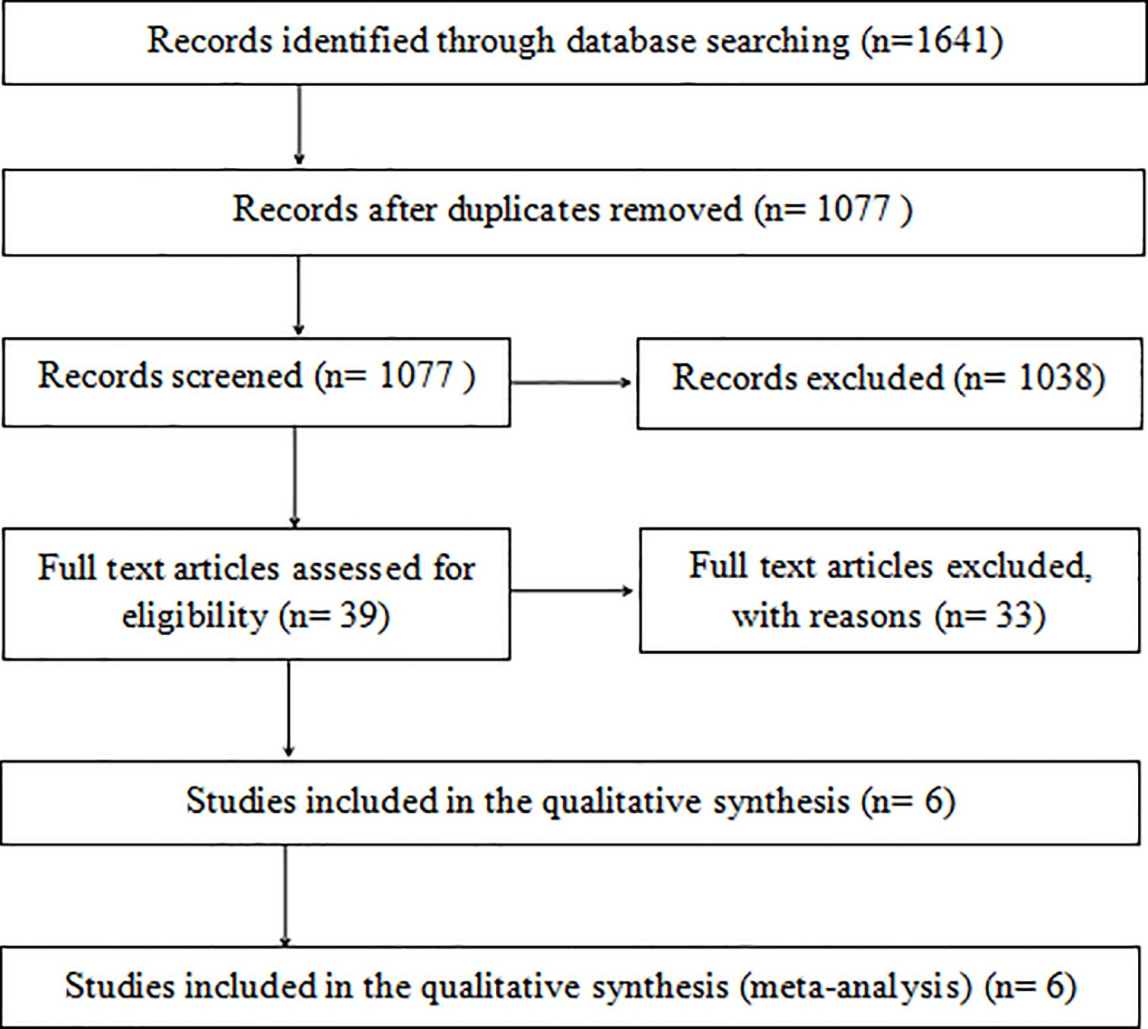
The flow chart of systematic review based on PRISMA statement.

The flow chart based on the PRISMA Statement [20] is shown in Fig 1. A total of 1641 records were preliminarily identified under our search strategy. Of these, 564 duplicated articles were eliminated. After screening the titles and abstracts, 39 potentially eligible studies were required to undergo the full-text analysis. Finally, 6 studies [21–26] that met all of the selective criteria were included in the current analysis. It’s worth pointing out that two [21,22] of the 6 articles were from a randomized, multicenter US Food and Drug Administration Investigational Device Exemption Study. Following in-depth discussion at the reviewers’ meeting, the consistent conclusion was that we could not rule out either of these 2 articles, because the evaluation indicators used in each article were different from those in the other article. Also, each of those indicators was exactly what our meta-analysis needed.

Characteristics of the included studies are summarized in Table 1. These 6 articles were published from 2005 to 2017 and included a total of 1093 patients, with sample numbers ranging from 48 to 577. Of these studies, 5 studies were RCTs [21–25] and 1 article [26] was an observational study. The mean age of patients ranged from 39.6 to 48.4. The follow-up time in the investigative and control groups ranged from 12 months to 60 months.

**Table 1:** Summary of characteristic of included studies.

Study	Design	No. of patients		Mean age		Gender		Follow up	Inclusion criteria	Outcome
		TDR	ALIF	TDR	ALIF	TDR	ALIF			
Blumenthal, 2005	RCT	205	99	39.6	39.6	M:113 F:92	M:44 F:55	24m	Symptomatic degenerative disc disease; single level at L4–L5 or L5-S1	Operative time, blood loss, hospital stay, patient satisfaction
Holt, 2007	RCT	205	99	39.6	39.6	M:113 F:92	M:44 F:55	24m	Symptomatic degenerative disc disease; single level at L4–L5 or L5-S1	Total adverse events, re-operation
Geisler, 2009	RCT	53	31	41.0	40.2	M:24 F:29	M:18 F:13	60m	Single level, symptomatic degenerative disc disease involvement L4–S1; TDR or ALIF no prior surgery	Operative time, blood loss, patient satisfaction,VAS, ODI
Gornet, 2011	RCT	405	172	39.9	40.2	M:205 F:200	M:86 F:86	24m	Degenerative disc disease; single-level, symptomatic involvement L4–S1	Operative time, blood loss, hospital stay, patient satisfaction,VAS, ODI, total adverse events, re-operation
Strube, 2016	RCT	23	25	47.3	48.4	M:10 F:13	M:10 F:15	12m	Degenerative disc disease; single level, symptomatic involvement L4–S1	ROM, VAS score, ODI score
Mattei, 2017	OS	30	50	40.5	45.3	M:15 F:15	M:25 F:25	12m	Degenerative disc disease; single-level symptomatic occurred at L4/L5 or L5/S1	Blood loss, VAS score, ODI score

OS = observational study; M = male; F = female; m = months; L = lumbar; S = sacral.

### Results of risk of bias assessment

According to the criteria of the Cochrane review group, the risk of bias of the included RCTs were rated as low to moderate, and the results are summarized in Table 2. Negative answers to the blinding could be found in almost all included studies. In fact, blinding of patients and care providers is often not feasible in the field of surgery. So, given the nature of the surgical research, we decided that we should not pay much more attention to the impact of blinding on the quality of the study. Moreover,only one observational study received a high quality score of 6 points (selection of cohorts: 2 scores, comparability of cohorts: 2 scores and assessment of outcome: 2 scores) based on NOQAS.

**Table 2:** Risk of bias assessment of included RCTs.

Type of bias	Criteria	Answers				
		Blumenthal, 2005	Holt, 2007	Geisler, 2009	Gornet, 2011	Strube, 2016
Selection	Was the method of randomization adequate?	Yes	Yes	Unsure	Yes	Yes
	Was the treatment allocation concealed?	Yes	Yes	Unsure	Yes	Yes
	Were the groups similar at baseline regarding the most important prognostic indicators?	Yes	Yes	Yes	Yes	Yes
Performance	Was the patient blinded to the intervention?	No	No	No	No	Unsure
	Was the care provider blinded to the intervention?	No	No	No	No	Unsure
	Were co-interventions avoided or similar?	Yes	Yes	Yes	Yes	Yes
Attrition	Was the drop-out rate described and acceptable?	Yes	Unsure	Yes	Yes	Yes
	Were all randomized participants analyzed in the group to which they were allocated?	Yes	Yes	Yes	Yes	Yes
Measurement	Was the outcome assessor blinded to the intervention?	No	No	No	No	Unsure
	Was the timing of the outcome assessment similar in all groups?	Yes	Unsure	Yes	Yes	Yes
Reporting	Are reports of the study free of suggestion of selective outcome reporting?	No	No	Yes	Yes	Yes
Other	Are other sources of potential bias unlikely?	Unsure	Unsure	Unsure	Unsure	Unsure

### Surgical data (TDR *versus* ALIF)

**Fig 2:**
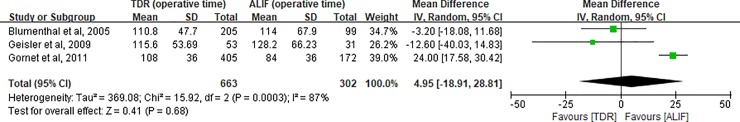
Forest plot for operative time. df = degrees of freedom, and IV = Inverse Variance.

**Fig 3:**
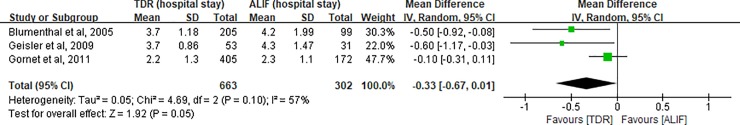
Forest plot for duration of hospitalization. df = degrees of freedom, and IV = Inverse Variance.

**Fig 4:**
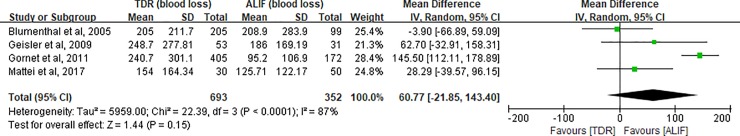
Forest plot for estimated blood loss. df = degrees of freedom, and IV = Inverse Variance.

Three RCTs [21,23,24] with sufficient data reported the operative time and duration of hospitalization of patients who were underwent TDR or ALIF. The pooled results shown that there was no significant difference in the operative time between TDR and ALIF groups [(Operative time: MD, 4.95; 95% CI, -18.91 to 28.81; P = 0.68; Fig 2); Duration of hospitality: MD, -0.33; 95% CI, -0.67 to 0.01; P = 0.05; Fig 3)]. Three RCTs [21,23,24] and one observational study [25] including a total of 1045 patients had the sufficient data on estimated blood loss. The synthetic results revealed that TDR did not show any difference from ALIF (MD, 60.77; 95% CI, -21.85 to 143.40; P = 0.15; Fig 4).

### Radiographic outcome (TDR *versus* ALIF)

Range of motion (ROM), as a good evaluation indicator after fusion had been reported in two studies [24,25]. Meta-analysis could not be performed due to insufficient data. Gornet et al [23] reported that the mean preoperative segmental ROM was 7°, and it increased at 12 and 24 months follow-up (9.4°and 9.5°, respectively). In the ALIF group, the mean ROM was less than 0.6°at any time after operation. However, a significant difference in postoperative lumbar mobility was observed in patients undergoing TDR (median ROM = 11.4°, interquartile range 4.95°), compared with that of patients in the ALIF group (median ROM = 0.4°, interquartile range 1.30°) [25].

### Clinical outcome (TDR *versus* ALIF)

**Fig 5:**
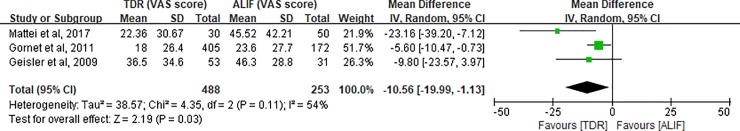
Forest plot for VAS score at the final follow-up. df = degrees of freedom, and IV = Inverse Variance.

**Fig 6:**
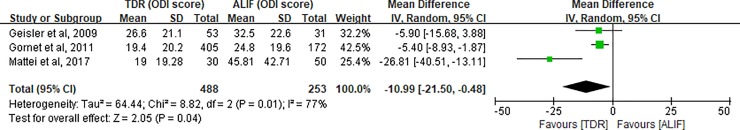
Forest plot for ODI score at the final follow-up. df = degrees of freedom, and IV = Inverse Variance.

**Fig 7:**
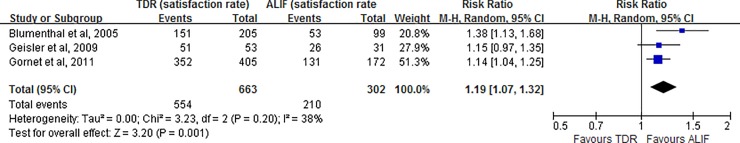
Forest plot for postoperative patient satisfaction. df = degrees of freedom, and M-H = Mantel-Haenszel.

In our meta-analysis, VAS, ODI score and postoperative patient satisfaction were considered as a category to assess the clinical curative efficacy. Each of the four clinical evaluation indicators was reported by three studies. The pooled results signified that there were significant differences in the VAS score at the final follow-up (MD, -10.56; 95% CI, -19.99 to -1.13; P = 0.03; Fig 5), in the ODI score at the final follow-up (MD, -10.99; 95% CI, -21.50 to -0.48; P = 0.04; Fig 6), and in the postoperative patient satisfaction (RR, 1.20; 95% CI, 1.11 to 1.30; P<0.00001; Fig 7).

### Complications and second surgery (TDR *versus* ALIF)

**Fig 8:**
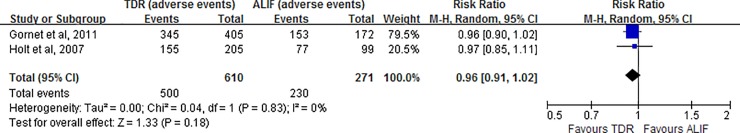
Forest plot for total adverse events. df = degrees of freedom, and M-H = Mantel-Haenszel.

**Fig 9:**
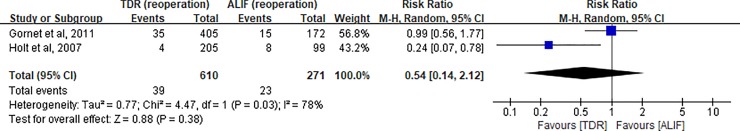
Forest plot for the number of re-operation. df = degrees of freedom, and M-H = Mantel-Haenszel.

Two RCTs [22,24] reported data on total adverse events and the second surgery. The pooled results reflected that there were no significant difference in the complications occurrence (RR, 0.96; 95% CI, 0.90 to 1.02; P = 0.22; Fig 8) or in the re-operation rates (RR, 0.54; 95% CI, 0.14 to 2.12; P = 0.38; Fig 9) between the two groups.

### Results of the GREAD system assessment

Based on the GREAD system, confidence in the estimates were high for patient satisfaction, moderate for operative time, duration of hospitalization and total adverse events, and low for blood loss, VAS, ODI score and re-operation (Table 3).

**Table 3:** Quality of evidence for outcome based on GREAD system.

Outcomes	No. of patients and trials	Study design (No.)	Risk of Bias	Inconsistency	Indirectness	Imprecision	Publication bias	Estimate of Effect (95% CI)	Confidence in Effect Estimates (GRADE)
Patient satisfaction	764 (3)	RCT (3)	No Serious	Not Serious	Not Serious	Not Serious	None detected	1.20 (1.11–1.30)	High ⊕⊕⊕⊕
Operative time	965 (3)	RCT (3)	No Serious	Not Serious	Not Serious	Serious^※^	None detected	4.95 (-18.91–28.81)	MODERATE ⊕⊕⊕O
Duration of hospitalization	965 (3)	RCT (3)	No Serious	Not Serious	Not Serious	Serious^※^	None detected	-0.33 (-0.67–0.01)	MODERATE ⊕⊕⊕O
Total adverse events	881 (2)	RCT (2)	Serious*	Not Serious	Not Serious	Not Serious	None detected	0.96 (0.90–1.02)	MODERATE ⊕⊕⊕O
Blood loss	1045 (4)	RCT (3) CC (1)	No Serious	Serious^†^	Not Serious	Serious^※^	None detected	60.77 (-21.85–143.4)	LOW ⊕⊕OO
VAS score	741 (3)	RCT (2) CC (1)	No Serious	Serious^†^	Not Serious	Serious^※^	None detected	-10.56 (-19.99—-1.13)	LOW ⊕⊕OO
ODI score	741 (3)	RCT (2) CC (1)	No Serious	Serious^†^	Not Serious	Serious^※^	None detected	-10.99 (-21.50—-0.48)	LOW ⊕⊕OO
Reoperation	881 (2)	RCT (3)	Serious*	Not Serious	Not Serious	Serious^※^	None detected	0.54 (0.14–2.12)	LOW ⊕⊕OO

*Selective outcome reporting among included studies.

^†^Different pooled estimates of effect between RCT and case-controlled study.

^※^Wide 95% CIs.

CC = case-controlled study; CI = confidence interval.

### Heterogeneity, sensitivity analysis

Meta-regression analyses were carried out in accordance with some covariates, including sample size, study design, and follow-up points; however, meta-regression outcomes did not detect the sources of heterogeneity (Table 4). A sensitivity analysis was performed by omitting one study (Blumenthal et al [21]). There was a significant difference between groups (MD = 0.42; 95% CI, 0.17–0.66)., which means that the results cannot be considered robust.

**Table 4:** Meta-regression analysis of potential sources of heterogeneity.

Factors	Coefficient	Standard error	P-value	95% confidence intervals
Sample size	0.544	0.606	0.435	-1.384, 2.472
Study design	1.094	0.362	0.802	-0.382, 3.135
Follow-up points	0.091	0.331	0.802	-0.962, 1.143

## Discussion

### Main findings

Five RCTs and one observational study involving 1093 cases were included in this meta-analysis. In the present study, we found that patients who underwent TDR had similar surgical data (operative time, intraoperative blood loss and hospital stay) and similar risk of postoperative adverse events compared with patients who underwent ALIF in the short term. However, the statistical differences in the results of comparison of clinical efficacy showed that patients who underwent TDR could achievebetter symptom relief and that their physical function improved.

### The results of the prior relevant meta-analysis

Several previous meta-analysis [11–13] comparing TDR to fusion for the treatment of lumbar disc degenerative disease have been published. A meta-analysis with 5 RCTs published in 2010 [11], reported that a significant statistical difference was only found in the postoperative patients’ satisfaction rate between both groups at 2-year follow-up. Although TDR revealed slight advantages in better functioning and pain remission, the pooled effects of multiple studies were not statistically significant. Importantly, TDR did not show significant superiority in the clinical outcomes at 5-year follow-up. Subsequently, two meta-analysis that included 6 and 7 RCTs under the same thesis were published in different peer-review journals by Wei et al [12] and Rao et al [13], respectively. Wei et al [11] reported that, the safety and efficacy of TDR during the 2-year follow-up were significantly better than that of lumbar fusion, but it was still not considered that TDR is superior to lumbar fusion. However, TDR was demonstrated significant superiorities in improved physical function, reduced pain and shortened duration of hospitalization in the Rao and colleagues’ manuscript [13].

Inconsistent findings prompted Ding et al [27] to conduct a systematic review of overlapping meta-analysis. After comparing 5 meta-analysis with the same topic, a paper by Jacobas et al [14] was rated to provide the best available evidence. They demonstrated that TDR was superior to lumbar fusion in patient satisfaction, ODI, VAS, pain, implant motion and subsidence [14]. Finally, the cautious conclusions that TDR was at least as safe and effective as lumbar fusion in the short term were presented in Ding and coleagues’ study [27]. However, the common defect in these meta-analysis that affected their findings is that different approaches of lumbar fusion for LDDD would result in different outcomes [28]. Circumferential fusion was the most used fusion approach in these meta-analysis. So, the results were largely influenced by this approach. In order to minimize the impact of confounding factors on the level of evidence, it is necessary to conduct a meta-analysis on the comparison of TDR and a specific surgical approach, such as ALIF, PLIF.

### The efficacy and safety of TDR

The efficacy of operation should be based primarily on patients’ safety. In the present study, surgical time, intraoperative blood, complications and re-operation rate were used to evaluate operative safety. The results were consistent with other studies [29]. Currently, most researchers believe that the early clinical efficacy of TDR is positive [30,31] on the premise of grasping strictly the operative indication. A variety of evaluation indicators were comprehensively analyzed in our study and the conclusion could be obtained that the efficacy of TDR is indeed better than that of ALIF for LDDD. As a new surgical technique, there were few mid- or long-term follow-up studies. Guyer et al [32] reported no statistical differences were found in clinical outcomes between TDR and ALIF, but patients who underwent TDR reached a statistically greater rate of part- and full-time employment and a statistically lower rate of long-term disability. Also, Zigler et al [33] believed that patients in the TDR group had significantly better improvement on some scales and were more satisfied about avoiding the stiffness of fusion than patients in the lumbar fusion group during the 5-year follow-up. Similar results were reported in the more than 15-year follow-up retrospective study [34].

The retention of lumbar segmental motion is considered to be the greatest advantage of TDR and the original intention of designing the artificial disc device. However, since the included studies didn’t provide sufficient data about ROM, we could not evaluate the studies by quantitative analysis. Descriptive qualitative analysis in our study showed that ROM after TDR was significantly better than ALIF. And whether it is with anyone of different fusion approaches or different artificial disc devices [35], the advantage of TDR in preserving postoperative ROM can be seen.

### Strengths and limitations

To our knowledge, this is the first meta-analysis of published clinical studies comparing the safety and efficacy of TDR and ALIF in patients with LDDD. Further, in our study, the application of the confidence in effect estimates can provide more institutional guidance for clinical decision-maker according to the GREAD system. However, this methodology has not been found in the previous studies with similar theses. In addition, under the strict methodology, significant superiorities of TDR in improved physical function, reduced pain and preserved range of motion were reported compared with ALIF in this meta-analysis. It is our hope that these results may be used to inform the clinical management of lumbar DDD, a condition that substantially influences quality of life in older adults.

However, this study was restricted by several limitations that require the cautious interpretation of the results. First, our meta-analysis that mainly focused on to investigating the safety and efficacy of TDR and ALIF for patients with LDDD had already excluded the influence of other fusion approaches on the results. However, the types of artificial intervertebral discs in the TDR group could not be stratified because of the limitation of relevant studies, so there may be an implementation bias. Moreover, although RCTs were the predominant studies included in this meta-analysis, blinding was not applied in these included studies, which may result in measurement bias. In addition, publication bias that can also affect the evidence level of conclusions couldn’t be well detected in the small meta-analysis with less than 10 included studies, because only publicly published studies were included in this meta-analysis and studies that reported the contrary finding may be difficult to publish. Also Additionally, language bias was found in our study. Finally, the result of the sensitivity analysis forced us to cautiously interpret the results of the study only.

Given that significant heterogeneity was present in the current study, we used the meta-regression analysis to test the factors that may be the sources of heterogeneity. Unfortunately, these specific covariates have proven to be not the factors influencing the safety and effectiveness of TDR. The cause for this finding has yet to be determined.

## Conclusions

In summary, this meta-analysis based on the current available studies shows that the efficacy of TDR is superior to that of ALIF in the short term. TDR may be an ideal alternative for selected patients with LDDD in the short term. However, the results of this study cannot suggest the use of TDR above the use of ALIF for lumbar spinal treatments only on the basis of short term results. Furthermore, we think that this study still has a certain clinical significance, although the limitations of this meta-analysis require us to be cautious about the present conclusions. Multicenter, well-designed, high-quality, large sample and long-term follow-up studies are needed to further evaluate the short- and long-term safety and efficacy of TDR comparison of ALIF or other fusion approaches in the treatment of LDDD.

## Supporting information

S1 File PRISMA 2009. Checklist.
(DOC)

S2 File Search strategy. (DOCX)
