# Supplementary material for: The short-term efficacy and safety of artificial total disc replacement for selected patients with lumbar degenerative disc disease compared with anterior lumbar interbody fusion: A systematic review and meta-analysis
Source: PLoS One. 2018 Dec 28;13(12):e0209660. doi: 10.1371/journal.pone.0209660 (PMC6310255; doi:10.1371/journal.pone.0209660)
Supplement: S2 File — (DOCX) [file pone.0209660.s002.docx]

**Appendix. Search strategy**

**Pubmed 2018/03/31——816 articles**

#1: Total disc replacement [Mesh terms]

#2: Artificial disc replacement [Mesh terms]

#3: Lumbar arthroplasty [All field]

#4: Lumbr disc implants [All field]

#5: Lumbar disc prostheses [All field]

#6: Prosthetic replacemen [All field]

#7: TDR [All field])

#8: #1 OR #2 OR #3 OR #4 OR #5 OR #6 OR #7

#9: Anterior lumbar interbody fusion [Mesh terms]

#10: Anterior fusion [All field]

#11: ALIF [All field])

#12: #9 OR #10 OR #11

#13: Unlimited to 31/03/2018 [Date - Publication]

#14: #8 AND #12 AND #13
